# Response to microtubule-interacting agents in primary epithelial ovarian cancer cells

**DOI:** 10.1186/1475-2867-13-33

**Published:** 2013-04-10

**Authors:** Ilenia Pellicciotta, Chia-Ping Huang Yang, Charis A Venditti, Gary L Goldberg, Shohreh Shahabi

**Affiliations:** 1 Department of Radiation Oncology, New York University College of Medicine, New York, NY 10016, USA; 2 Department of Obstetrics & Gynecology and Women’s Health, Division of Gynecologic Oncology, Montefiore Medical Center, the Albert Einstein College of Medicine and the Albert Einstein Cancer Center, Bronx, New York, NY 10461, USA; 3 Department of Molecular Pharmacology, Albert Einstein College of Medicine, Bronx, NY 10461, USA; 4 Department of Obstetrics, Gynecology, and Reproductive Biology, Danbury Hospital, 24 Hospital Avenue, Danbury, CT 06810, USA

**Keywords:** Primary ovarian cancer cells, Microtubule-interacting agents, Cell cycle analyses, Chemosensitivity, Epothilone B

## Abstract

**Background:**

Ovarian cancer constitutes nearly 4% of all cancers among women and is the leading cause of death from gynecologic malignancies in the Western world. Standard first line adjuvant chemotherapy treatments include Paclitaxel (Taxol) and platinum-based agents. Taxol, epothilone B (EpoB) and discodermolide belong to a family of anti-neoplastic agents that specifically interferes with microtubules and arrests cells in the G2/M phase of the cell cycle. Despite initial success with chemotherapy treatment, many patients relapse due to chemotherapy resistance. *In vitro* establishment of primary ovarian cancer cells provides a powerful tool for better understanding the mechanisms of ovarian cancer resistance. We describe the generation and characterization of primary ovarian cancer cells derived from ascites fluids of patients with epithelial ovarian cancer.

**Methods:**

Chemosensitivity of these cell lines to Taxol, EpoB and discodermolide was tested, and cell cycle analysis was compared to that of immortalized ovarian cancer cell lines SKOV3 and Hey. The relationship between drug resistance and *αβ*-tubulin and p53 status was also investigated.

**Results:**

All newly generated primary cancer cells were highly sensitive to the drugs. *αβ*-tubulin mutation was not found in any primary cell lines tested. However, one cell line that harbors p53 mutation at residue 72 (Arg to Pro) exhibits altered cell cycle profile in response to all drug treatments. Immortalized ovarian cancer cells respond differently to EpoB treatment when compared to primary ovarian cancer cells, and p53 polymorphism suggests clinical significance in the anti-tumor response in patients.

**Conclusions:**

The isolation and characterization of primary ovarian cancer cells from ovarian cancer patients’ specimens contribute to further understanding the nature of drug resistance to microtubule interacting agents (MIAs) currently used in clinical settings.

## Background

Ovarian cancer constitutes the fifth most common cause of cancer deaths among women in the Western world and is the leading cause of death from gynecologic malignancies. Standard first line adjuvant chemotherapy treatments include Paclitaxel (Taxol) and platinum-based agents. Taxol, epothilone B (EpoB) and discodermolide belong to a family of anti-neoplastic agents that stabilizes microtubules and arrests cells in the G2/M phase of the cell cycle. Despite initial success with chemotherapy treatment, many ovarian cancer patients will relapse due to chemotherapy resistance. Consequently, there is a strong impetus to investigate the molecular and biological characteristics of ovarian cancer to develop more effective diagnostic and therapeutic strategies.

To date, established immortalized ovarian cancer cell lines have served as a useful substrate to study this disease. However, immortalized tumor cell lines undergo many manipulations during their development that may limit our ability to translate experimental results from cell lines to actual ovarian disease in the clinical setting [1-4]. The establishment of primary ovarian cancer cells *in vitro* provides a powerful tool for better understanding the mechanisms of ovarian cancer resistance. Malignant epithelial tumors account for about 85% of ovarian cancers. Ascites is commonly present in women with epithelial ovarian cancer, and it is associated with advanced-stage disease. Paracentesis is usually performed to alleviate pain and discomforts, and it may collect 2–4 liters of ascites fluid. We describe here a modified protocol to isolate and grow *in vitro* cultures of primary ovarian cancer cells derived from ascites fluids of patients with histologically confirmed epithelial ovarian cancer. Tumor cells can be easily isolated from ascites and they may survive for days or months either dividing or not dividing before eventually dying, or may divide repeatedly requiring sub-culturing or splitting. We have established a series of primary ovarian cancer cell cultures from the ascites of patients with ovarian carcinoma and have characterized them while still at an early stage in culture. We have studied their growth characteristics, histochemical properties, and used them as a model to study *in vitro* sensitivity to microtubule interacting agents. In particular, we have studied cell cycle analyses of primary ovarian cancer cells treated with Taxol, EpoB and discodermolide, and compared it to that of immortalized ovarian cancer cell lines SKOV3 and Hey - commonly used for ovarian cancer studies. Although it has been widely reported that Taxol exerts cytotoxic effects to primary ovarian cancer cells [5], this is the first time to the best of our knowledge, that the sensitivity to EpoB and discodermolide is described.

Human malignant tumors are characterized by abnormal proliferation resulting from alterations in cell cycle-regulatory mechanisms. The regulatory pathways controlling cell cycle phases include several oncogenes and tumor suppressor genes that display a range of abnormalities with potential usefulness as markers of evolution or treatment response in ovarian cancer. It is known that MIAs alter cytoskeleton equilibrium in tumor cells and consequently affect cell proliferation and drug resistance. Therefore, tubulin sequence in the newly generated primary tumor cells has been assessed in this study. Most importantly, because p53 tumor suppressor protein plays a major role in modulating cellular response to therapeutic agents and it is implicated in the late stage of malignant transformation, p53 mutants’ analysis has been evaluated in ascites-derived primary tumor cells.

Our data demonstrate that the primary ovarian cancer cells we have developed provide a direct tool for the study of fresh primary tumor cells derived from patients with ovarian cancer allowing further characterization of the disease through *ex vivo* chemosensitivity assays and gene profiling.

## Results

### Establishment of ovarian cancer cell cultures from patients’ ascites

**Figure 1:**
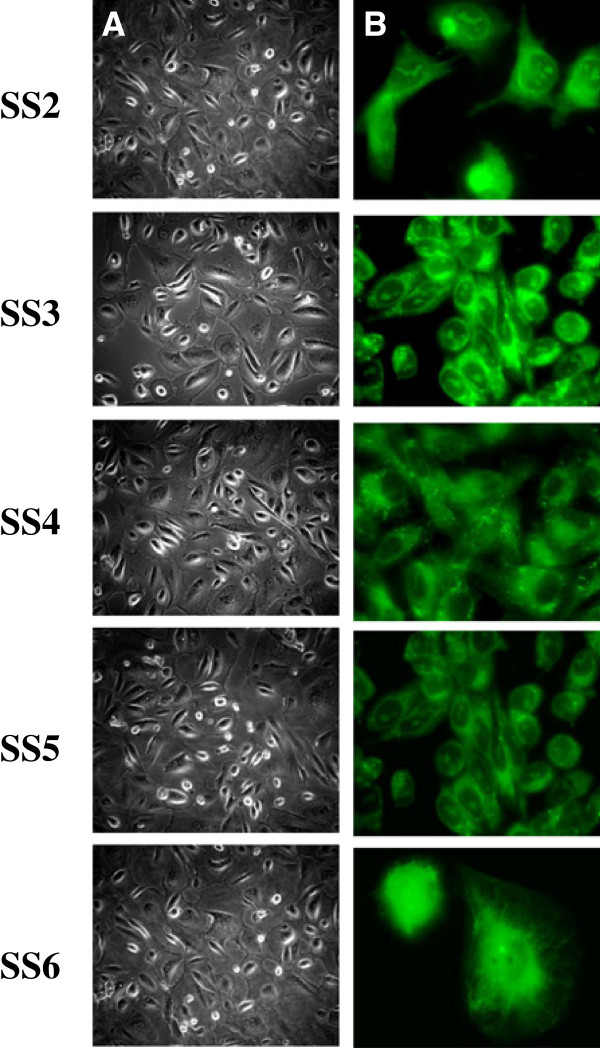
**Primary cultures of epithelial ovarian cancer cells isolated from five patients’ ascites fluid.** (**A**) Confluent monolayer of primary cells illustrating typical epithelial cobblestone morphology. (**B**) Immunofluorescent detection of cytokeratin expression in primary ovarian cancer cells demonstrating the epithelial origin. An area representative of the entire image was selected.

Peritoneal ascites specimens were collected at the time of surgery from 25 patients with histologically confirmed epithelial ovarian cancer. All patients were scheduled to undergo laparatomy for diagnostic and/or therapeutic purposes or clinically indicated paracentesis. Primary ovarian cancer cells were isolated from approximately 250 ml of patients’ ascites fluid and cultured with growth media as reported in Material and Methods. Ovarian cells were found as single cells or small grape-like clusters in the ascites fluids – this would conveniently avoid the need for mechanical or enzymatic desegregation. This report describes the isolation and characterization of primary ovarian cancer cells from five different patients (SS2 to SS6). After six days of *in vitro* culture (Figure 1A) primary cells appear as confluent monolayer illustrating typical epithelial cobblestone morphology. To confirm the epithelial origin, immunofluorescent detection staining of cytokeratin expression was performed on all primary cells in culture (Figure 1B).

### Sensitivity assay to Taxol, epothilone B and discodermolide

To characterize the drug sensitivity profiles, the newly established primary ovarian cancer cell cultures were tested in cytotoxicity assays with Taxol, EpoB and discodermolide. Cells developed from patients’ ascites have been exposed to scalar concentrations of each drug. The values reported in Table 1 show the quantitative measure of the half maximal (50%) inhibitory concentration - IC_50_- of the drugs. SS3, SS4 and SS5 displayed the highest sensitivity to all drugs, with IC_50_ values ranging from 0.17-0.19 nM for Taxol, 0.15-0.34 nM for EpoB and 1.1-1.5 for discodermolide. IC_50_ values relative to immortalized ovarian cancer cell lines SKOV3 and Hey are also reported. As shown in Table 1, the IC_50_ values vary widely among the various cell lines when treated with Taxol, EpoB and discodermolide. We noted that SS2 cells demonstrated the lowest sensitivity and highest resistance to Taxol compared to all other cell lines in this series. Conversely, SS6 cells demonstrated the lowest sensitivity and highest resistance to EpoB and discodermolide compared to all other cell lines. These variations of IC_50_ values among the freshly generated primary cells lines demonstrate that primary cells lines indeed display individual profiles inherent for sensitivity and resistance to various microtubule stabilizing agents (MSAs).

**Table 1:** **IC**_**50 **_**values for primary and immortalized ovarian cancer cells upon drug treatment**

	**Taxol**	**Epothilone B**	**Discodermolide**
SS2	1.10 nM	0.05 nM	3.20 nM
SS3	0.19 nM	0.23 nM	1.14 nM
SS4	0.19 nM	0.15 nM	1.45 nM
SS5	0.17 nM	0.34 nM	1.18 nM
SS6	0.54 nM	2.51 nM	6.28 nM
SKOV3	10.0 nM	3.2 nM	52.1 nM
HEY	2.5 nM	0.9 nM	39.3 nM

IC_50_ values were determined after 72 hours (for immortalized cells SKOV3 and HEY) and 12 days (for primary ovarian tumor cells) of incubation at 37°C with the indicated drugs. Concentration range for primary tumor cells: Taxol and EpoB: 0.1-50 nM, discodermolide: 0.2-100 nM; for Hey and SKOV3 cell lines: Taxol and EpoB: 0.5-250 nM; discodermolide: 1–500 nM.

### Cell cycle kinetics of primary ovarian cancer cells following exposure to microtubule interacting agents

**Figure 2:**
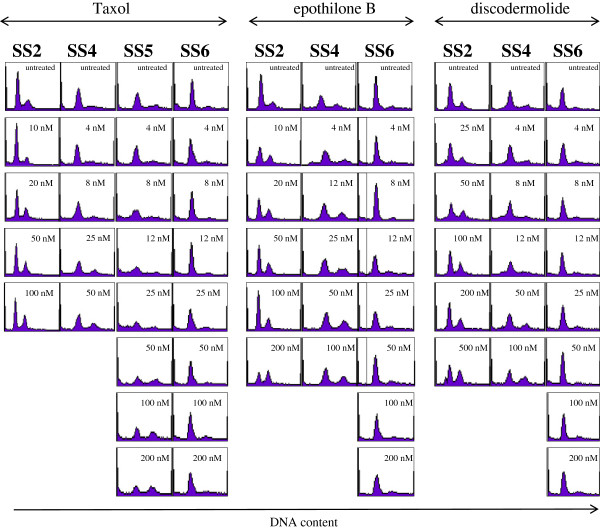
**Characterization of cellular response to MSAs.** Ascites-derived primary ovarian cancer cells were treated with Taxol, EpoB or discodermolide at the indicated doses. Cells were analyzed for cellular proliferation and DNA content was measured by FACS analysis using propidium iodide after 72 hrs. One representative sample of 2–3 replicates with similar results is shown.

In order to better comprehend the mechanisms of difference sensitivity of primary ovarian cancer cells to MSAs, cell cycle analyses was performed on all five ascites-derived ovarian cancer cell lines. Figure 2 shows propidium iodide staining performed to evaluate the effect of Taxol, EpoB and discodermolide on modulating cell cycle. Ovarian cancer cells generated from patients’s ascites were treated with scalar concentrations of the drugs (4 to 200 nM) and cell cycle was evaluated 72 hours after treatment. However, because the number of cells in each patient’s ascites varied substantially, treatment with all drugs was not performed for each specimen. Figure 2 (left panel) shows the characterization of the cell cycle in response to Taxol treatment. Data relative to the cell cultures SS2, SS4, SS5 and SS6 are reported. After treatment with Taxol, an overall decrease in cells arrested in the G0/G1 phase is observed in SS2, SS4 and SS5 specimens. Concurrently there is an increase in cells arrested in the G2/M phase of the cell cycle. Although the cellular response of these three cell specimens is overall consistent, the extent and the time of the response is slightly different; SS5 cells in fact require a treatment with 50 nM to be arrested in the G2/M phase while SS2 and SS4 respond to lower doses of the drug. Interestingly, SS6 cells display a very different response to Taxol treatment. SS6 cells were treated with 4, 8, 12, 25, 50, 100 and 200 nM Taxol: no modulation of the cell cycle can be observed after 72 hrs of treatment. These data suggest that the ascites-derived SS6 primary ovarian cancer cell specimen might be resistant to Taxol treatment, and cell cycle arrest cannot be induced in this case at the drug treatment conditions used here.

To evaluate the ability of other MSAs to interfere with cell cycle of primary ovarian cancer cells, SS2, SS4 and SS6 specimens were selected for treatment with EpoB and dicodermolide. Cell cycle is modulated by treatment with both drugs similarly to Taxol-treated cells. SS2 and SS4 specimens’ cell cycle is arrested by nanomolar concentrations of EpoB and disodermolide: 10 nM and 12 nM of EpoB induce cycle arrest at the G2/M phase of SS2 and SS4 cells respectively (Figure 2, middle panel). A higher dosage of 50 nM of discodermolide is necessary to induce a similar pattern of cell cycle modulation in SS2 and SS4 cells (Figure 2, right panel). Similarly to Taxol, cell cycle of SS6 specimen is not arrested or modified to any extent by treatment with EpoB and discodermolide. The results also show that DNA content in the cells derived from this specimen does not reveal any changes in cell cycle distribution following drug treatment. This finding suggests that SS6 specimen is highly resistant to Taxol, EpoB and discodermolide *in vitro*.

### αβ-tubulin and p53 mutational assay

It is known that expression of mutant tubulin and p53 proteins, results in reduced sensitivity to cell death induced by chemotherapeutic drugs and MIAs specifically. To understand why different cell specimens exhibit different sensitivity to same drugs, we isolated RNA from the five ascites-derived ovarian cancer cells, followed by cDNA preparation and analyzed the sequence of *αβ*-tubulin and p53. Table 2 shows that no *αβ*-tubulin modification was detected in the primary ovarian cancer cells. Sequence variations in p53 were identified in the RNA from two specimens. Specifically, a variant in exon 8, which confers an amino acidic substitution of arginine to proline at codon 72 was identified in SS3 and SS6 specimens. Interestingly, SS6 is also the only ovarian cancer cell line showing resistance to the treatment with EpoB, Taxol and discodermolide as shown in the cell cycle analyses reported in Figure 2. This finding suggests a possible correlation between the observed p53 mutation and cell sensitivity to the chemotherapeutic drugs tested in this study.

**Table 2:** αβ-tubulin and p53 mutational assay

	**αβ-tubulin**	**p53**
SS2	W	W
SS3	W	Arg72Pro
SS4	W	W
SS5	W	W
SS6	W	Arg72Pro

W: wild type; Arg72Pro: sequence variant in p53 exon 8, which confers an amino acid substitution of arginine to proline at codon 72.

### Cell cycle kinetics of immortalized cell lines after treatment with EpoB

**Figure 3:**
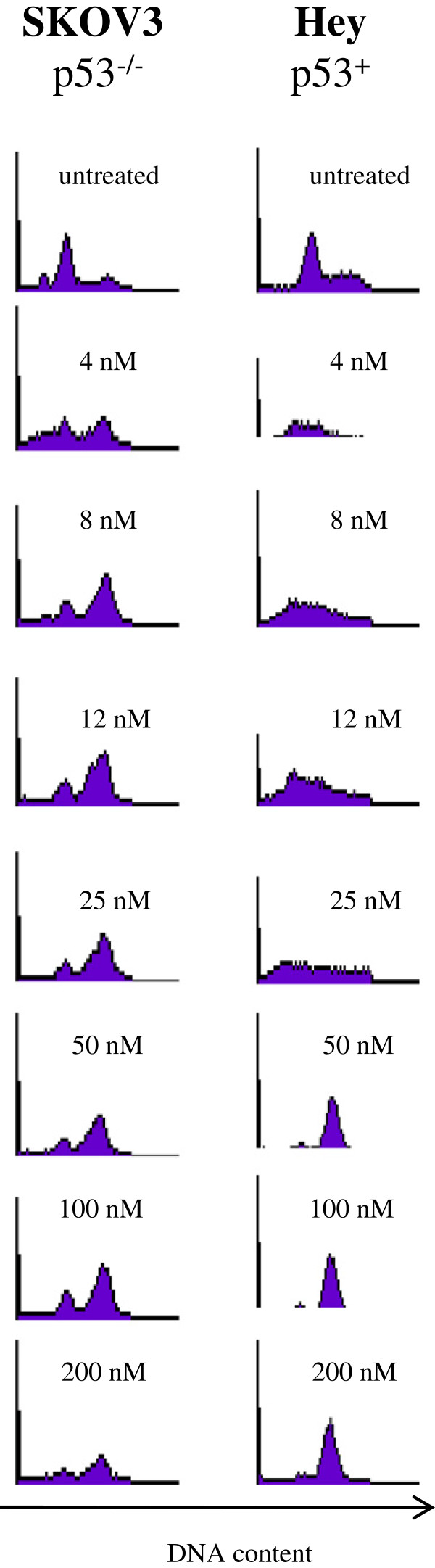
**Effect of EpoB on p53**^**−/− **^**cell line (SKOV3) versus a p53**^**+ **^**cell line (Hey).** Cells were treated with scalar concentrations of EpoB (4nM to 200nM) and propidium iodide staining was performed after 24 hrs of treatment to assess cell cycle response to the drug.

To further investigate the correlation of p53 status with drug sensitivity, we compared the effects of EpoB treatment on the cell cycle of two immortalized ovarian cancer cell lines: a p53^−/−^ null cell line, SKOV3, versus a p53^+^ wild type cell line, Hey. Figure 3 shows the cell cycle analysis performed after 24 hrs of treatment with various concentration of EpoB. As little as 4 nM of EpoB is sufficient to arrest approximately 50% of the p53^−/−^ cells SKOV3, while a higher dosage of 50 nM is necessary to noticeably arrest cell cycle of the majority of the p53^+^ cells Hey. Interestingly, when cell cycle response to EpoB of the primary ovarian cancer cell line SS6 bearing mutation on p53 (Figure 2B) is compared to the response of the immortalized p53^−/−^ null cell line SKOV3 also carrying p53 mutations (Figure 3), an opposite effect can be observed. In fact, while SS6 primary cells are not affected by EpoB treatment, SKOV3 cell cycle is arrested by as low as 4 nM of the drug. Additionally, the primary cells SS4 and the immortalized cell line Hey, despite both carrying wild-type p53 sequences, show different sensitivity to EpoB, as SS4 cell cycle is arrested by lower concentrations compared to Hey cells. Altogether these data show that primary ovarian cancer cells freshly isolated from patients can be differently affected by chemotherapeutic drugs compared to immortalized cell lines that are commonly used as *in vitro* models for human ovarian cancers. We therefore can speculate that primary human ovarian cancer cell lines generated as we described may provide clinically relevant models more suitable for investigation of the *in vitro* biological characteristics of ovarian cancers, which in turn may lead to the discovery of new therapies for these tumors.

## Discussion

In this report we describe the isolation and characterization of primary human ovarian cancer cell lines derived from untreated patients diagnosed with histologically confirmed epithelial ovarian cancer. We confirmed that it is feasible to develop primary ovarian cancer cells from patients’ ascites fluids, and that these cells can be used to characterize mechanisms of action of microtubule-stabilizing agents (MSAs), Taxol, epothilone B and discodermolide. Furthermore we report that p53 and tubulin mutational analysis can also be assessed in the newly generated primary tumor cells.

A simple protocol has been designed to isolate single cells and small grape-like clusters of ovarian cancer cells found in the ascites fluids. In general, after a patient specimen was plated *in vitro*, the first cells to attach on the plastic of the flask were the fibroblasts; macrophages also thrived in tissue culture. These cell populations could divide only a finite number of times, until the cancer cells took over the culture. Ovarian cancer cells grew *in vitro* as to acquire a monolayer distribution with the typical epithelial cobblestone morphology (Figure 1). Importantly, no mechanical or enzymatic disaggregation was involved in our protocol for the establishment of cell culture, and the least manipulation of the cells was performed. Therefore the original properties as well as the physical features of the tumor cells were maintained. While most ovarian carcinoma cell lines commercially available have been passaged for prolonged periods and may therefore have lost certain characteristics, the cell lines described herein were studied at early passage numbers, and stored in liquid nitrogen to maintain a source of cells*.* Despite tumor heterogeneity and unknown source of the tumor cells found in the ascites (primary tumor versus metastasis), these cells can be considered as a close representation of the tumors in their natural state, and can be used for further characterization such as genotyping, drug sensitivity assays, and establishment of xenograft *in vivo* models. In this study, ovarian cancer cell lines isolated as we described, provided a direct and straightforward tool to investigate the sensitivity to commonly used anti-neoplastic agents belonging to the MSA family. Specifically, we evaluated cytotoxicity of Taxol, EpoB and discodermolide by exposing primary ovarian cancer cells to scalar concentrations of each drug. We observed that all cell lines were affected by the treatment and it was feasible to define the IC_50_ for each drug. Because these cells are highly selected, their behavior may differ significantly in regard to growth characteristics and response to drug. Within the five cell lines reported here, IC_50_ values varied upon the same drug treatment. In particular, SS2 showed the highest IC_50_ to Taxol and SS6 showed the highest IC_50_ values to EpoB and discodermolide. Additionally, SS6 showed no cell cycle modulation after treatment with Taxol, EpoB and discodermolide, suggesting a mechanism of resistance in the specimen derived from that patient.

In order to further characterize the cytotoxic response to chemoterapuetic drugs, different doses of Taxol, EpoB and discodermolide were tested (see Table 1). We found that the primary cell lines we established were able to respond to each drug and that cell cycle is extensively modified by the drugs. Studies performed with immortalized ovarian cancer cell lines show that microtubule interacting agents (MIAs) generally induce modification of microtubule network and G_2_/M arrest in proliferating cells. After 72-hour incubation with cytotoxic concentrations of the drugs, the primary tumor cell lines we generated showed modifications of the cell shape (data not shown). Cell cycle analysis indicated that exposure to the drugs decreased the proportion of cells in the S phase and increased the proportion in the G0/G1 and/or G2/M phases. However, the dose-dependent sensitivity to each drug may vary significantly, and SS6 cell cycle distribution was not affected by any drugs (Figure 2). Immortalized cell lines are commonly used in *in vitro* models to study the effects of chemotherapeutic drugs in ovarian cancer. We compared cell cycle modifications induced by EpoB on immortalized ovarian cancer cell lines SKOV3 and Hey to those of freshly isolated primary tumor cells. SKOV3 and Hey showed a cell cycle pattern that is very different from that described for the primary tumor cells isolated from patients (Figure 3). Specifically, the primary tumor cells grew much slower than the immortalized cells. It was observed that when the same number of cells was plated, primary tumor cells required approximately 10–12 days to achieve 60-70% confluency, whereas immortalized cells only needed 3 days to reach 95% confluency. Moreover, a different cell cycle response is observed between the two cell lines, probably due to their different p53 status: p53^−/−^ (SKOV3) and p53^+^ (Hey). It is known that patients with mutant p53 ovarian tumors were found more responsive to paclitaxel-based chemotherapy [6], indicating that p53 status might differentially affect drug response in different cell types. The introduction of mutations in drug-targeted proteins is one of the mechanisms affecting response to anti-cancer drug treatments. Although the molecular basis of drug sensitivity and resistance is complex, the p53 tumor suppressor protein appears to play a major role in modulating cellular response to therapeutic agents. p53 tumor suppressor protein is the most commonly mutated protein in diverse cancers and it is implicated in the late stage of malignant transformation [7]. An extensive number of studies have shown somatic p53 mutations to be a predictor of response to treatment in ovarian cells resistant to cisplatin [8]. Generally, the presence of wild-type *p53* in tumor cells correlates with a good clinical response to drug therapy. Mutations in the p53 gene are extremely frequent in ovarian cancer and they vary among the histological types. Not surprisingly, loss of apoptotic functions appears to be a major cause of resistance to cytotoxic drugs and p53 mutations are observed in more than 50% of advanced ovarian carcinomas [9]. Presence of wild-type p53, on the other hand, does not necessarily ensure a chemosensitive phenotype, and a clinical study has underscored this very convincingly reporting that, among the group of chemoresistant ovarian tumors, 37% had wild-type *p53* and 63% had mutant *p53*. The study also reports that loss of p53 function has been associated with the lack of response to high-dose cisplatin in ovarian cancer patients [10]. To investigate the reasons for different drug response observed in our freshly generated primary ovarian cancer cells, a p53 mutational study was assessed and we found that two out of the five cell lines tested, carried a homozygous missense mutation at codon 72 in the exon 8 of the p53 gene. This mutation confers an amino acidic substitution of arginine to proline (Figure 3). The Arg72 form has a high risk of developing into other forms of cancer compared to the Pro72 form and it induces apoptosis markedly more than the Pro72 variant [11]. These differences may influence cancer risk or treatment [12]. Interestingly, one of the two cell lines carrying the codon 72 mutation showed no cell cycle modifications after treatment with Taxol, EpoB and discodermolide. This finding suggests that correlations between the p53 mutation observed in the primary cells and their sensitivity to chemotherapeutic drugs cytotoxicity can be speculated.

The significance of codon 72 polimorphysm in the resistance to chemotherapeutic drugs and patient prognosis is still controversial [13-15]. Although the polymorphism is balanced, it varies with latitude and race, and is maintained at different allelic frequencies across the population [16], it is suggested to result in a drastically altered biological and biochemical behavior of p53 *in vitro*. Its clinical relevance is correlated to differences in apoptosis susceptibility to cytotoxic drugs and the response and survival to radiochemotherapy [13,17,18]. Codon 72 mutations have been associated with urothelial [19], thyroid [20], colorectal carcinomas [21] and chronic myeloid leukemia [22], whereas homozygosity for arginine 72 was associated with advanced lung cancer [23]. In Italian breast cancer patients, the retention of an arginine allele was correlated with a reduction of survival in one study [24]. Furthermore, p53 protein encoded by the arginine allele appears to be more susceptible to HPV-E6 protein-induced degradation [25]. p53 polymorphism can partially explain the heterogeneity of cell cycle response observed in our primary cell lines after drug treatment. We hypothesize that: (i) different p53 mutants may have specific properties against different chemotherapeutic drugs, as the expression of a specific p53 mutant results in resistance to one drug but does not affect the sensitivity against another drug; (ii) although the codon 72 mutants tend to confer resistance to anticancer drugs, it is not an universal phenomenon and depends on the mutation and drug used; (iii) cell type–specific effects of the codon 72 mutants may be observed. Our data generated from analysis of drug response in primary tumor cells, in addition to the data published from others, highlight the significance of evaluating the role of p53 mutations in conjunction with the polymorphic status before the therapeutic regimen is decided upon.

Because MSA alter cytoskeleton equilibrium in tumor cells and, because abnormal microtubule dynamics impair mitotic spindle function and inhibit normal cell proliferation [5,26-29]*,* the relationship between MSAs and drug resistance in our primary ovarian cancer cells has also been evaluated. Factors that influence microtubule dynamics include tubulin mutations that affect interactions between protofilaments or binding of regulatory proteins of different phosphorylation states. These tubulin mutations could result in microtubules with increased dynamics or decreased stability [30,31]*.* It has been suggested that an alteration in microtubule dynamics may be involved in Taxol or EpoB resistance/dependence phenotype [28,32]*.* Specifically, mutations in β-tubulin have been found in a variety of Taxol and EpoB resistant cells [33], where they are responsible for microtubule stability decrease. Some of these resistant cells may become more sensitive to microtubule-destabilizing drugs, such as vinblastine and colchicine [26,34]. In the light of these observations, we tested αβ-tubulin sequence in the five primary ovarian cancer cell lines described herein (Table 2). No αβ-tubulin sequence mutations were found, however it remains to be assessed whether different tubulin mutants exist and to what extent these variants affect drug resistance. In addition, MSAs may alter the expression and phosphorylation of microtubule associated proteins that may influence the dynamic stability of microtubules.

Although p53 and tubulin mutants may explain the heterogeneity observed in the response of primary ovarian cancer cells to the chemotherapeutic drugs used in this study, several additional biological reasons can be hypothesized. The majority of our data indicated that exposure to the drugs arrested cell cycle of primary tumor cells on the G2/M phases, although a clear heterogeneity in the cell cycle patterns among the different specimens can be observed. Generally, increased cytotoxicity was reported with higher drug concentrations, yet a different dose–response relationship was observed for each cell line and each individual drug. It would be interesting to assess whether the dose–response patterns vary for combined drug exposures and if the combination is more active than each individual drug. The protocol we have generated for isolation of tumor cells form ascites fluids does not distinguish from primary tumor and its metastases in the same patient. This may lead to different sensitivity profiles to the same drug or to different drugs as well. Tumor heterogeneity, therefore, reduces the reliability of the test in making clinical decisions.

The differences in cell cycle response to EpoB observed in primary tumor cells relatively to immortalized cell lines Hey and SKOV3, can be attributed to the cross-talk between the tumor microenvironment and malignant epithelial cells, that can largely influence apoptotic response. Primary cells isolated from patients’ ascites have been recently in contact with tumor microenvironment and received specific signals that immortalized cells cultured *in vitro* for longer time, may have lost. Additionally, it appeared from our studies and studies from others that, even in supposedly morphologically homogeneous cell lines and similar fresh human tumors, subpopulations displaying different patterns of response to similar cytotoxic agents may be identified. In this scenario it is likely that neither the response to a single drug nor to drug combinations could be predicted for a particular tumor specimen other than by direct testing. This underscores the need to characterize an individual patient’s chemosensitivity profile before drug treatment. Advances in cell culture methodologies have made it possible to establish a variety of human carcinoma cell lines from surgical tissues and biopsy specimens. Therefore, despite tumor heterogeneity and uncertain origin of ascites tumor cells, the isolation and establishment of primary tumor cells derived from patients and the analyses of their response to various chemotherapeutic drugs would be a most useful tool in making clinical decisions.

## Conclusions

To overcome drug resistance in patients, we need methods that reliably measure individual tumor sensitivities. The results described in this work show that we have generated a simple protocol to isolate and establish primary ovarian cancer cells from ascites fluids. We demonstrated that these primary ovarian cancer cells can be used for *in vitro* studies to provide information on the effect of microtubule interacting-agents such as Taxol, EpoB and discodermolide *in vitro* thus delineating whether a pattern of resistance exists. Furthermore we report that p53 and tubulin mutational analysis can also be assessed in the freshly generated primary tumor cells. These primary tumor cells can be used for a variety of additional studies including genotyping and establishment of xenograft *in vivo* models, and specifically we have shown and discussed the relevance they may play in understanding the biology of resistance to chemotherapeutic drugs.

## Methods

### Isolation of primary ovarian cancer cells from ascites

With Institutional Review Board approval, physicians in the division of gynecologic oncology at Montefiore Medical Center obtained ascites specimens from 25 patients with ovarian cancer at the time of surgery. Institutional informed patient consent was obtained for this study. Patients were scheduled to undergo laparatomy for diagnostic and/or therapeutic purposes or clinical indicated paracentesis. Approximately 250 ml of ascites was obtained from each patient. In each cell culture flask 20 ml of fresh ascites fluid was mixed with 20 ml of growth medium RPMI1640 media, supplemented with 10% FBS, 100 unites/ml penicillin and 100 μg/ml streptomycin. Regular passaging of the cell lines was carried out in complete culture medium conditioned with 20% of their own medium and 10% of autologous filtered ascites. After 4–5 passages, the cell cultures were completely free of fibroblasts and mesothelial cells. Aliquots of cells were frozen in liquid nitrogen, at different passage numbers, in 5% dimethyl sulfoxide (DMSO) in FCS. Cells were used for phenotypic characterization and drug resistance analyses after three days of culture (passage 1–2) and were fully confluent after six days of culture. Cells were isolated from all 25 patients in the study cohort, and subsequently cultured and frozen in liquid nitrogen for future studies. Six out of the 25 patient specimens were selected for the microtubule agents studies described here.

### Immortalized cell lines and microtubule interacting agents

The human ovarian cancer cell lines SKOV3 and Hey were grown at 37°C in RPMI 1640 containing 10% FBS. Taxol, EpoB and discodermolide were obtained as previously reported [26,35].

### Immunofluorescence analysis of tumor cell epithelial origin

Primary cells isolated from ascites fluid were analyzed to assess epithelial origin. A total of 1 × 10^6^ cells were washed with PBS and non-specific binding was blocked with 5% BSA in PBS for 1 h at 37°C. Cells were washed with PBS and incubated for 20 minutes on ice in the dark with anti-cytokeratin mAb, followed by incubation with FITC-labeled secondary anti-mouse antibody. The cells were washed with PBS with 2% fetal calf serum (Sigma-Aldrich, St. Louis, MO) and cytospun into glass slides and analyzed with a microscope.

### Cytotoxicity assay

A methylene blue-based cytotoxicity assay was developed from previous research to study the drug resistance profiles of the primary ovarian cancer cells [36,37]. Approximately 5,000 cells were seeded into each well in a 96-well plate and allowed to settle for 16 h, and drugs were added to the first well of the plate and serial diluted to subsequent wells. After 72 hrs of incubation for Hey and SKOV3 cells and 12 days for primary tumor cells, the medium was discarded, and 100 μl of a methylene blue solution (0.5% in ethanol:water; 50%, v/v) were added to each well. One hour later, unbound methylene blue was washed off with water and bound stain was solubilized by the addition of 1 ml of 1% SDS solution. The plates were stirred gently on a rotator for 1 h at room temperature, and the absorbance in each suspension was read at 630 nm in a spectrophotometer. The staining in the control well was taken as 100%, and the IC_50_ were defined as the drug concentrations that inhibited the cell growth by 50%. Drug concentration range for primary tumor cells: Taxol and EpoB: 0.1-50 nM, discodermolide: 0.2-100 nM; for Hey and SKOV3 cell lines: Taxol and EpoB: 0.5-250 nM; discodermolide: 1–500 nM.

### Flow cytometry cell cycle analysis

After treatment with Taxol, EpoB and discodermolide, cells were collected by centrifugation and washed twice with ice-cold PBS. Cells were resuspended and fixed in 70% ethanol at 4°C for 1 h. After centrifugation, cells were washed twice in PBS and resuspended in 1 ml of PBS containing 20 μg/ml of propidium iodide and 5 Kunitz units of DNase-free RNase A. Samples were incubated at 37°C for 30 min and the cell cycle distrubution was determined by flow cytometry using a FACSCalibur (Becton-Dickinson, Franklin Lakes, NJ). Two to three replicates were performed for each sample.

### Tubulin and p53 sequence analysis

Total RNA was prepared from cells as described [38] and reverse transcribed to cDNA. For RT-PCR and sequencing four overlapping sets of primers were designed based on the published sequence (GenBank accession no. AF070561) and previously published primer sequences [34]. Total RNA was isolated using Total RNA isolation reagent (ABgene, Rochester, NY) and contaminating DNA removed with RNase-free DNase I treatment (Boehringer Mannheim, Indianapolis, IN) for 30 min at 37°C. Total RNA (1 μg) was reverse transcribed and the cDNA used for RT-PCR. PCR-amplified products were purified using the QIAquick gel extraction kit (Qiagen, Valencia, CA). The amount of cDNA was quantified, and ∼200 ng of cDNA, with 6 pmol of either primer, forward or reverse, was sequenced using an automated DNA sequencing system (ABI Prism). The accession numbers are as follows: TP53, AAD28535; Tubulin alpha chain, Q71U36; Tubulin beta chain, Q13509.

## Competing interests

The authors declare that they have no competing interests.

## Authors’ contributions

IP analyzed and interpreted the data and composed the manuscript. CPHY revised the manuscript, contributed to experimental methodologies and helped analyzing the data. CV participated in the data analysis and helped to draft the manuscript. SS conceived and designed the study, performed the experiments, and revised the manuscript. GG contributed to conception and design and has given the final approval of the version to be published. All authors read and approved the final manuscript.
